# A comprehensive mental health care program for women with polycystic ovary syndrome: protocol for a mixed methods study

**DOI:** 10.1186/s12978-018-0488-5

**Published:** 2018-03-13

**Authors:** Fatemeh ZareMobini, Ashraf Kazemi, Ziba Farajzadegan

**Affiliations:** 1 Student Research Center, Faculty of Nursing and Midwifery, Isfahan University of Medical Sciences, Isfahan, Iran; 2 Reproductive health Department, Faculty of Nursing and Midwifery, Isfahan University of Medical Sciences, Isfahan, Iran; 3 Community medicine Department, Faculty of medicine, Isfahan University of Medical Sciences, Isfahan, Iran

**Keywords:** Intervention program, Psychological health, Polycystic ovary syndrome (PCOS), Needs, Reproductive health, Mental health promotion

## Abstract

**Background:**

Psychological health is related to the management of polycystic ovary syndrome (PCOS) and is an essential component of self-efficacy and enjoying a healthy lifestyle. Need assessment and plans to improve the psychological health of these women provides significantly valuable information to design an advantageous intervention program to reach that goal. Therefore, this study will conduct to improve the psychological health for women with PCOS through a designed comprehensive care.

**Methods:**

This is an exploratory mixed method study using consecutive qualitative-quantitative methods, including three consecutive phases. In the first phase, a qualitative study will be conducted to assess their needs and will design a care protocol for improving mental health of women with PCOS. Participants in this phase will select by purposive sampling method and data will collect using semi-structured interviews by taking notes at same time. Data will analyze using conventional content analysis method. At second phase, according the information obtained from previous phase and a literature review a comprehensive program to mental health care will be proposed. Then multidisciplinary team will review and finalize it according to priorities. The third phase will follow a quantitative approach using quasi-experimental study with two groups to measure the effectiveness of this program on the women’s psychological health.

**Discussion:**

Designing a program based on a qualitative study and a review article and updated evidences can lead to improving of these women’s psychological health and quality of life. Consequently, we expect to show that mental health program provided by a multidisciplinary team improves reproductive outcomes while at the same time being cost-effective in women with PCOS.

## Plain English summary

Psychological health is related to the management of polycystic ovary syndrome (PCOS) and is an essential component of self-efficacy and enjoying a healthy lifestyle. The present study provides strong information and data regarding the needs and strategies for improving the psychological health in women with PCOS. This is an exploratory mixed method study using consecutive qualitative-quantitative methods, including three consecutive phases. In the first phase, a qualitative study will be conducted to assess their needs and will design a care protocol for improving mental health of women with PCOS. Participants in this phase will select by purposive sampling method and data will collect using semi-structured interviews by taking notes at same time. Data will analyze using conventional content analysis method. At second phase, according the information obtained from previous phase and a literature review a comprehensive program to mental health care will be proposed. Then multidisciplinary team will review and finalize it according to priorities. The third phase will follow a quantitative approach using quasi-experimental study with two groups to measure the effectiveness of this program on the women’s psychological health. Therefore, designing a program based on a qualitative study and a review article and updated evidences can lead to improving of these women’s psychological health and quality of life. Consequently, we expect to show that mental health program provided by a multidisciplinary team improves reproductive outcomes while at the same time being cost-effective in women with PCOS.

## Background

Polycystic Ovarian Syndrome (PCOS), the most common endocrine disorder in women of reproductive age; its prevalence has been shown to be 6 to 18% based on Rotterdam diagnostic criteria [1–3]. Clinical presentation includes oligomenorrhea, obesity, acne, reproductive disorders and hirsutism [4–6]. Moreover, because of impaired glucose tolerance, insulin resistance, type II diabetes, an abnormal increase in blood lipids and high blood pressure, patients with PCOS are at increased risk of cardiovascular disease [7, 8]. Also, due to its chronic nature and variety of its symptoms, PCOS affect the patients’ different aspects of life. Studies suggest that in addition to physical problems [8], and emotional disorders [9] their quality of life is impacted by disorder [10], and PCOS leads to psychological problems [9–18]. Pcos creates an important psychological burden throughout the life-time of these women [9, 10, 19]. It has been reported that 57% of women with polycystic ovary syndrome have at least one mental disorder [20]. High levels of androgens, acne, infertility and fear of it, hirsutism, high body mass index, low body image, sexual problems and coping with the disease impair the mental health of them [20, 21].

Mental health is fundamental to good health and wellbeing and influences social and economic outcomes across the lifespan [22]. Also, mental health is an important part of public health [23] and it is one of the most important indicators of the quality of health care in society [24]. Many chronic illnesses have mental health impacts [25]. In PCOS, symptoms and co-morbidities increase the risk of adverse psychological health consequences. While, mental health is particularly related to the management of polycystic ovary syndrome and is an essential component of self-efficacy and enjoying a healthy lifestyle [20, 25]. Lifestyle modification is the priority in the management of polycystic ovary syndrome, since small changes in these women’s lifestyle and a balanced weight, can improve symptoms, and increase ovulation and improve fertility [6, 26]. However, mental disorders including depression or anxiety reduce these critical components and prevent of lifestyle modification and subsequently the disease will show negative clinical outcomes [25, 27]. Considering the importance of mental health, some researchers suggest that there has been little attention paid to the mental health of women with PCOS, and it is necessary to evaluate and manage the mental health among this group of women [28]. Therefore, this exploratory mixed method study will carry out to define and explore their needs and demands to provide a standard patient oriented protocol for women with PCOS.

## Methods

This is an exploratory mixed method study using consecutive qualitative-quantitative methods, including three consecutive phases. In the first phase, a qualitative study will conduct and the conclusion resulted from this phase will analyze using a conventional content analysis method to determine the needs and strategies related to improving mental health among the women with PCOS.

At second phase, according the information obtained from previous phase and a literature review a comprehensive program to mental health care will be proposed. Then multidisciplinary team will review and finalize it according to priorities.

**Fig. 1:**
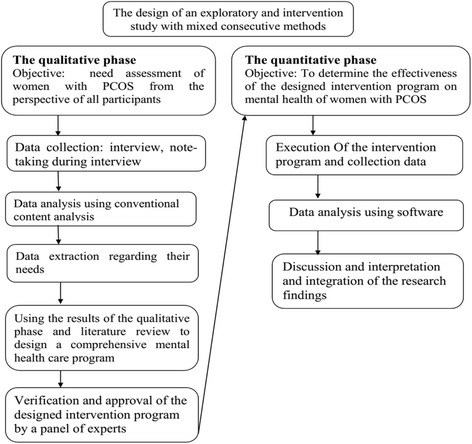
The general design of the study

The third phase will follow a quantitative approach using quasi-experimental study with two groups to measure the effectiveness of this program on the women’s psychological health. The general design of the study is presented in Fig. 1.

### Phase I: Qualitative study

In this phase of the study, needs and strategies related to the mental health of women with PCOS will determine using semi-structured deep interviews, which will analyze using conventional content analysis method. The study environment included women’s health clinics, clinics for endocrinology, dermatology, laser centers, and infertility centers affiliated to the Isfahan University of Medical Sciences. All interviews will conduct in a private and contented place. Health worker interview will be held in their office.

#### Participants

The participants of this study include women with PCOS based on Rotterdam diagnostic criteria with the maximum diversity of the disease range, duration of their illness, age, education, marital status, social class, job and their spouses. Also, health service providers with an experience in providing health care and treatment services to women with PCOS including gynecologists, dermatologists, endocrinologists, nutritionist, reproductive health specialists, general practitioners, midwives, psychiatrists, psychologists, social workers also will participate in the study with informed consent. Participants will select through purposive sampling, and the interviews will conduct with those who had the inclusion criteria after receiving their informed consent. We continue the interviews until data saturation, and the interviews will stop if data saturation occurs.

### Inclusion criteria

Iranian women with a confirmed diagnosis of PCOS, who have competency to take, participate in interview, no history of cancer or chronic and psychological disorder. Health care providers with at least one year experience for caring this women.

### Qualitative data collection method

In this phase, data will gather through open and semi-structured interviews as well as taking notes on the spot. The following actions will be performed for ethical consideration, this study was confirmed by the Ethical Committee of Isfahan University of Medical Sciences, explanations will provided for the participants regarding the objectives of the study. Written and verbal consent will obtain from the participants for recording their interviews. Time schedule, length and place of interview will selected with participant preference.

#### The method for qualitative data analysis

Conventional content analysis will apply for data analysis and interpretation.

#### Accuracy and reliability of the qualitative data

To assure the trustworthiness of the findings, the four criteria credibility, dependability, confirmability, transferability will apply [29].

### Phase II: Designing the intervention protocol

In the second phase of the study, a comprehensive mental health care program will prepared based on need assessment and approaches extracted from the qualitative study and literature review to improve the mental health of women with PCOS, and it will validate by an experts panel. Literature review will conduct using narrative review method included searching library sources (reference books, thesis, and dissertations) as well as electronic database search to access the existing knowledge related to issue. In this phase, all studies with key words including; PCOS, AND “mental health” plus PCOS, AND “mental health improvement” that were published in English or Persian, between 2006 and 2017 will review. Databases of PubMed, ScienceDrect, Web of Science, Cochrane Library, Ovid, Scopus, ProQuest, Magiran, Embase, SID Database Will search.

### Holding an multidisciplinary team

At this step, to achieve experts’ consensus, the technique of RAM (Rand Appropriateness Method) will use [30]. The process is as follow: using the results of the qualitative study and reviewing the literature, creating and sending a list of health care services to multidisciplinary team. The multidisciplinary team will grade each health care service considering benefits to harm ratio on the scale of 1 to 9, where 1 means the expected harms are higher than the benefits, and 9 means its expected benefits greatly outweigh the harm. Finally, each item in the list will classify as “Good,” “indeterminate” or “not good” based on the ratings and scores. In the second round, a meeting of all multidisciplinary team will be held, including experts in endocrinology, reproductive health, dermatology, nutritionist, obstetrics and gynecology, midwives, psychologists and psychiatric nurses. During the meeting, these experts discuss the prioritization of strategies while taking into account the conditions and the time limitation for the intervention process.

### Phase III: Quantitative study

In this step a randomized controlled clinical trial will design to assess the efficacy of prepared protocol. Participants randomly will allocate into two groups. In experimental group we will implement our designed protocol, and control group will not receive any intervention, but they follow as same as experimental group. For ethical issues all educational material will offer them at the end of study. Mental health status will assess as a desired outcome.

#### Study environment and population

Ambulatory clinics for gynecology, endocrinology, dermatology, laser therapy centers, and infertility centers affiliated to the Isfahan University of Medical Sciences will consider as a research environment.

#### Study sample

Target populations for study are all women with confirmed diagnosis of PCOS based on Rotterdam diagnostic criteria.

#### Sampling method

Convenience sampling’s method will apply to select the participants.

#### Inclusion criteria

Inclusion criteria are women aged between 15 and 49 years, Iranian nationality, consent to participate in the study, ability to understand questions or ability to read and write, not being in stressful situations such as immigration, death of loved ones and financial problems in the past 6 months, not participating in any other clinical trials at the same time, no severe mental disorders that requires to be under medical treatment or hospitalization.

#### Exclusion criteria

The exclusion criterion is not completing the intervention for any reason such as severe life stress, the death of the spouse or immigration and so on.

### Data collection method

The instrument for collecting mental health data is the GHQ-28.This questionnaire has 4 subscales including somatic symptoms, anxiety/insomnia, social dysfunction, and severe depression. The cut of point of the questionnaire is 23; which means that a score less than 23 signifies mental health and 23 and above signifies mental health problem and the subject needs to be referred to a psychiatric.

#### Data analysis:

We will apply X^2^ and independent T test to analysis our data by using SPSS version 21.

## Discussion

Various studies emphasized on the necessity of mental health evaluation and management among women with PCOS [5, 28, 31–33] and suggested that addressing the mental health problems should be a routine part of the health evaluation in clinical setting [14, 16, 28, 33, 34]. Also, studies explain that since there has been little attention paid to the psychological effects of this disease, it is necessary to plan for comprehensive care, including psychological aspects of PCOS and determine the needs of women with this disease [31, 34]. The present study provides strong information and data regarding the needs and strategies for improving the mental health in women with PCOS. Therefore, designing a program based on a qualitative study and by considering the context, and a review article and updated evidences can lead to enhancement of these women’s mental health and quality of life. It can also reduce their medical and treatment costs. We suppose this program has capacity to integrate into the professionally health care guidelines, so that it can help medical and health care providers pay attention to the important role of the mental health alongside treatment methods of the physical and mental problems of women with PCOS, especially during reproductive age. The strategies of this program could be important and cost effective, and therefore we hope that the success of such a program is a step forward in improving their health status and quality of life.

## Abbreviations

GHQ-28: General Health Questionnaire-28
PCOS: Polycystic Ovarian Syndrome
RAM: Rand Appropriateness Method
